# Everybody's Page

**Published:** 1888-05-26

**Authors:** 


### Everybody's Page.

In the middle ages, when the various saints of the calendar were worshipped as minor deities, the belt of St. Guthlac was believed to be a sovereign remedy for headache.

Coal-miners, masons, and persons who work in materials which give rise to dust, are all subject to serious lung disease from the penetration of these particles into the air-cells.

High mountain air is very conducive to lung exercise, because the air being rarified, more must be taken into the chest to satisfy the requirements of the blood in the matter of oxygen.

The heart will beat twenty-four to thirty-six hours, or even longer, after it is taken out of the body, if it be supplied with blood or some other nutrient fluid. The heart of a shark has been known to beat for three or four hours on the taffrail of a ship.

It seems some bacteria have the power of feeding on the living tissue, and of waging war on the cells of the tissues. This has been described by a Russian, who, in studying some of the lower crustacea, observed a battle going on between the blood cells and bacteria. Sometimes the little bacteria attacked the blood corpuscle and defeated it; sometimes the blood corpuscle attacked and defeated the bacteria. This led to the conclusion that living cells are capable sometimes of destroying bacteria, whilst at other times the bacteria flourish, and lead to special diseases.

For invalids, young children, and old people, white fish is of great value. The whiting is the most delicate, and least likely to disagree; it is sometimes called "the chicken of the sea." The haddock, flounder, sole, and plaice are all easy of digestion. Turbot has more flavour, but is less digestible. Several eminent authorities—Pavy, Sir Risdon Bennett, and others—state that cod is much less digestible than other white fish. This opinion the late Sir Robert Christison repeatedly and emphatically said was incorrect, and that it is only when cod is tough and woolly that it should be avoided.

A serious source of danger in the air is the presence of sewer-gas. In old houses the waste and soil pipes are usually combined in one single tube, which commonly runs inside the walls. When such pipes have become corroded and leaky, the gases pass from them into the house. In all modern houses of good construction the waste and soil pipes are kept separate, and they are placed outside the walls, with careful arrangements for trapping and ventilation. But there are houses of another kind, run up, not for the unwary inhabitant to live *in*, but for the "jerry builder" to live *by*. In such houses we often find that the inmates suffer from headaches resembling those caused by defective ventilation, from low forms of sore throat, and from troublesome inflammations of any wounds that may happen to be present.

The late Dean Ramsay told a story of a Highland drunkard, on whom the minister was trying to impress the fact that whisky was a very bad thing. "Aye, aye," said Donald, "it's a bad thing whisky, specially bad whisky." Whisky maybe dangerous to some men, but adulterated whisky is poison to all men. There was a case at Greenock some years ago in which a man paid 2*s.* 7*d.*, for a bottle labelled "Finest Old Highland Whisky." That was found to be mixed with sulphuric acid to such an extent as to be dangerous to health. About the same time thirty samples of whisky were taken in various parts of Glasgow; only two of these were pure, the others contained wood naphtha, oil of vitriol, turpentine, sulphate of copper, shellac, and chlorine water. These are terrible things for Donald to reflect upon.

Russian physicians claim to have had great success in the treatment of alcoholic habit by means of nitrate of strychnine in doses of one-sixtieth of a grain. The drug is said not only to relieve the immediate effects of alcoholic excess, but in many cases to destroy the craving for stimulants.

An article called flexible glass is now made by soaking paper of proper thickness in copal varnish, thus making it transparent, polishing it when dry, and rubbing it with pumice stone. A layer of soluble glass is then applied and rubbed with salt. The surface thus produced is said to be as perfect as that of ordinary glass.

Fish is one of the foods specially calculated to repair the waste of the body and to build up new tissue; and that, while the general principle applies to fish as to other foods—that living too exclusively on it produces various disorders—its qualities as a food certainly entitle it to rank higher than it seems to do at present in popular estimation.

The word alcohol is derived from the Arabic, and was first applied to an impalpable powder used by the Egyptian ladies to tinge their hair and eyebrows. Afterwards it came to be applied, in the sense of a subtle essence, to the fluid obtained by distillation from wine, which also received the name of spirit of wine.

An American manufacturer of sugar-coated pills added to the attractions of an exhibit of his product in London an ingenious piece of mechanism, which might have been intended to represent the pharmacist of the future. It was in the form of a cabinet provided with a series of knobs or buttons, each inscribed with the name of some malady for which a remedy might be asked. The customer puts a coin into a slit and presses the button, calling for the remedy he requires, when immediately a drawer flies out containing the article sought. This automatic dispenser, of course, makes no mistakes. If the customer accidentally presses the wrong button, he alone is responsible for the error. Is this really what we are coming to?

Many invalids, when their food is limited to milk diet, come to loathe it very heartily, but they would find milk jelly a pleasant variation on the form in which it is usually offered to them. Here is a recipe: Heat one quart of milk with one pound of sugar, and when the sugar is dissolved continue the heat, at a boiling temperature, for about ten minutes. Now cool it well, and then add, slowly stirring, a solution of one ounce of gelatine in a cupful of water. Next add the juice of three or four lemons and three wine-glasses full of wine, brandy, or other liquor. Set the glasses containing the mixture in a cold place, so that the contents may set. It is necessary to have the milk quite cold before the other ingredients are added, as it would otherwise curdle

The reputation of Æsculap water as a mild but effective aperient continues to grow. Constipation, as one of the minor ills of town life, occupies a prominent place. The natural and sufficient action of the bowels every day often makes all the difference between happiness and misery. Some witty person disposed of Mr. Mallock's book and argument "Is life worth living?" in a single sentence. "It depends upon the liver" said the wit. No doubt it does to a very large extent, on the liver and the pocket at any rate. Æsculap water acts very gently upon the liver, and it does not worry afterwards. We do not say that every household should possess it, because there are here and there households so preternaturally and unpardonably healthy that the doctor and all his infinite resources are utterly despised. But every household where livers are known to be, even occasionally, troublesome, would do well to have a dozen bottles of Æsculap always in the cellar. It is one of the pleasantest medicines in the world for women and children if properly manipulated and converted into lemonade with lemon juice and sugar.

